# Meta- analysis and meta-regression analysis of the associations between sex and the operative outcomes of carotid endarterectomy

**DOI:** 10.1186/s12872-015-0029-x

**Published:** 2015-05-09

**Authors:** Thomas Luebke, Jan Brunkwall

**Affiliations:** grid.411097.a000000008852305XDepartment of Vascular and Endovascular Surgery, University Hospital of Cologne, Kerpener Str. 62, 50937 Cologne, Germay

**Keywords:** Carotid endarterectomy, Complications, Meta-analysis, Meta-regression, Sex, Gender

## Abstract

**Background:**

Subgroup analyses from randomized controlled trials (RCT) of carotid endarterectomy (CEA) for both symptomatic and asymptomatic carotid stenosis suggest less benefit in women compared to men, due partly to higher age-independent peri-operative risk. However, a meta-analysis of case series and databases focussing on CEA-related gender differences has never been investigated.

**Methods:**

A systematic review of all available publications (including case series, databases and RCTs) reporting data on the association between sex and procedural risk of stroke and/or death following CEA from 1980 to 2015 was investigated. Pooled Peto odds ratios of the procedural risk of stroke and/or death were obtained by Mantel-Haenszel random-effects meta-analysis. The I^2^ statistic was used as a measure of heterogeneity. Potential publication bias was assessed with the Egger test and represented graphically with Begg funnel plots of the natural log of the OR versus its standard error. Additional sensitivity analyses were undertaken to evaluate the potential effect of key assumptions and study-level factors on the overall results. Meta-regression models were formed to explore potential heterogeneity as a result of potential risk factors or confounders on outcomes. A tria sequential analysis (TSA) was performed with the aim to maintain an over- all 5 % risk of type I error, being the standard in most meta- analyses and systematic reviews.

**Results:**

58 articles reported combined stroke and mortality rates within 30 days of treatment. In the unselected overall meta-analysis, the incidence of stroke and death in the male and female groups differed significantly (Peto OR, 1,162; 95 % CI, 1.067-1.266; *P* = .001), revealing a worse outcome for female patients. Moderate heterogeneity among the studies was identified (*I*^2^ = 36 %), and the possibility of publication bias was low (*P* = .03). In sensitivity analyses the meta-analysis of case series with gender aspects as a secondary outcome showed a significantly increased risk for 30-day stroke and death in women compared to men (Peto OR, 1.390; 95 % CI, 1.148-1.684; *P* = .001), In contrast, meta-analysis of databases (Peto OR, 1.025; 95 % CI, 0.958-1.097; *P* = .474) and case series with gender related outcomes as a primary aim (Peto OR, 1.202; 95 % CI, 0.925-1.561; *P* = .168) demonstrated no increase in operative risk of stroke and death in women compared to men.

**Conclusions:**

Metanalyses of case series and databases dealing with CEA reveal inconsistent results regarding gender differences related to CEA-procedure and should not be transferred into clinical practice.

## Background

Carotid endarterectomy (CEA) has been shown to be a more effective therapeutic option compared to the best medical treatment alone in the prevention of ischemic stroke, with acceptably low perioperative (30-day) stroke and death rates [1–4]. The subgroup analyses of NASCET (North American Symptomatic Carotid Endarterectomy Trial), ACAS (Asymptomatic Carotid Atherosclerosis Study), and ECST (European Carotid Surgery Trial) suggested that CEA may not be as efficacious in women as it is in men [5, 6] and that women might have higher risk of perioperative adverse events compared to men [1, 2]. It has been speculated, that the lower magnitude of benefit in women was due partly to a slightly higher operative risk in women as compared to men combined with the lower natural history risk of stroke in women [5–7]. However, it is not clear whether these subgroup analyses can be transferred to a non-trial setting because the CEA trials had specific inclusion and exclusion criteria. Moreover, even among patients who are eligible for randomisation, it is known from other trials that there are systematic differences between patients who are recruited and those who are not [8] and trial recruitment tends to be most selective in women [9]. Whereas a considerable body of literature challenges the overall benefit of CEA in unselected women compared to men, other studies on larger CEA databases suggested no substantial gender differences [10, 11]. Therefore it is crucial to determine whether the gender related differences of operative risks in women in the trials of CEA are also present in routine clinical practice. Since only operative mortality, rather than the risk of stroke and death, is recorded in the large-scale statewide or national reports of routinely collected data on outcome after CEA [12–15], a meta-analysis of all available publications (including case series, databases and RCTs) published during 1980–2015 that reported the perioperative risk of stroke and death following CEA by gender was performed.

## Methods

### Information sources and search strategy

We conducted PubMed (1950 to present), EMBASE (1980 to present), and Cochrane Central Register of Controlled Trials searches using the Medical Subject Headings terms *endarterectomy*, *carotid*, *stents*, and *carotid stenosis* and combining them with key terms associated with sex (eg, *sex*, *gender, men, male, women, and female*) and the word *risk*. The last search was run in January 2015. A secondary search consisted of manual scrutiny of the reference lists of review articles, meta-analyses, and original studies identified by the electronic searches to find other eligible trials. There was no language restriction for the search.

### Eligibility criteria

All published studies reporting 30-day (or similar) perioperative risk of stroke and/or death following CEA for symptomatic or asymptomatic stenosis, which stratified their results according to patient sex either as the main objective of the study or as a substudy were considered.

Studies were included if they fulfilled the following criteria:

1. The numbers of combined strokes and/or deaths occurring within 30 days of CEA (or similar time period) were reported.
2. The risks of stroke and/or death were defined, or calculable, per operation.
3. Operative risks were reported according to sex of the patients.

Studies were excluded if:

1. They concerned carotid surgery for non-atherosclerotic disease.
2. They included patients undergoing bilateral simultaneous CEA and did not report data separately on patients undergoing unilateral procedures.
3. They included patients undergoing synchronous CEA and coronary artery bypass grafting and did not report data separately on patients undergoing CEA only.
4. They concerned the risks of surgery in a specific sex but did not report data on the other sex.

### Data collection

The data sought included (1) study characteristics (year of publication, patient recruitment period, number of patients or procedures); (2) baseline demographic and clinical characteristics of the patients (age, sex, hypertension, diabetes mellitus, coronary artery disease, peripheral artery disease, dyslipidemia, smoking status, and symptomatic or asymptomatic carotid disease); (3) procedural characteristics (type of anesthesia, use of shunt, and type of CEA [primary or redo CEA]); and (4) outcome parameters, as defined above.

### Quality assessment

Study quality was quantified with the Newcastle-Ottawa Scale [16] for case–control observational studies. The Jadad scale was applied for the assessment of RCTs [17].

### Statistical analysis

#### Overall analyses

Meta-analyses were performed to calculate the overall relative odds of death, and combined stroke and death according to sex by the Mantel-Haenszel method. The Peto method for odds ratios (ORs) [18, 19] was used for studies with few events. To counterbalance random effects of the different studies, such as variabilities of baseline characteristics, the summary estimates of Peto ORs was applied. Intention-to-treat meta-analysis was performed in line with recommendations from the Cochrane Collaboration and the Preferred Reporting Items for Systematic Reviews and Meta- analyses Statement [20] using standard software (Comprehensive Meta-Analysis 2.0 software, Biostat, Englewood, NJ).

#### Sensitivity analyses

Analyses were also performed separately for trial (RCTs) and non-trial populations (databases and case-series) and, among non-trial populations, for studies in which the effect of sex on operative risk was the primary focus (primary aim) of the study versus those where it was reported as a subanalysis, usually along with other risk factors (secondary aim). Where the data were reported, the proportions of asymptomatic patients patients amongst females versus males were determined also. In order to analyse those studies separately with a contemporary and adequate best medical treatment and surgical procedure a cut-off was set for the last ten years, analysing studies published before the year 2004 versus those published after the year 2004. The difference between the estimates of the subgroups was analysed according to tests for interaction [21]. *P* < .05 indicates that the effects of treatment differ between the tested subgroups.

#### Proof of heterogeneity and bias

Heterogeneity was assessed using the I^2^ statistic [22]. I^2^ is the proportion of total variation observed between the trials attributable to differences between trials rather than sampling error (chance), with *I*^*2*^ < 25 % considered low and *I*^*2*^ > 75 % considered high. Potential publication bias (ie, bias resulting from the greater likelihood of publishing favourable results) was assessed with the Egger test and represented graphically with Begg funnel plots of the natural log of the OR versus its standard error [23, 24].

#### Meta-regression

A full meta-analysis random-effect approach to the regression had been used, where studies are weighted by a combination of their within-study variance and the degree of heterogeneity. In detail, meta-regression models were formed to explore potential heterogeneity as a result of changes in practice over time and to evaluate the effect of age and other risk factors or potential confounders on outcomes (coronary artery disease, peripheral artery disease, arterial hypertension, diabetes mellitus, dyslipidemia, smoking status, symptom status). We used residual maximum likelihood to estimate the additive (between-study) component of variance 2 for the metaregression analysis. Bootstrap analyses were performed using a Monte Carlo permutation test for metaregression using 10 000 random permutations [25].

#### Trial sequential analysis

Cumulative meta-analysis of trials is at risk for producing random errors because of few data and repetitive testing of accumulating data, and the information size requirement analogous to the sample size of a single optimally powered clinical trial may not be met [26, 27]. In order to quantify the risk for random errors a trial sequential analysis (TSA) was performed The underlying assumption for TSA is that significance testing is performed each time a new trial is published. The TSA depends on the quantification of the required information size. In this context, the smaller the required information size is, the more lenient the TSA is, thus the more lenient the criteria are for statistical significance [26, 27]. The TSA was performed with a desire to maintain an over- all 5 % risk of type I error, being the standard in most meta- analyses and systematic reviews, and we calculated the required information size (ie, the meta-analysis information size needed to detect or reject an intervention effect of a 20 % [or 15 %] relative risk increase [RRI] with a 20 % risk of type II error and power of 80 %) [26, 27].

## Results

**Fig. 1:**
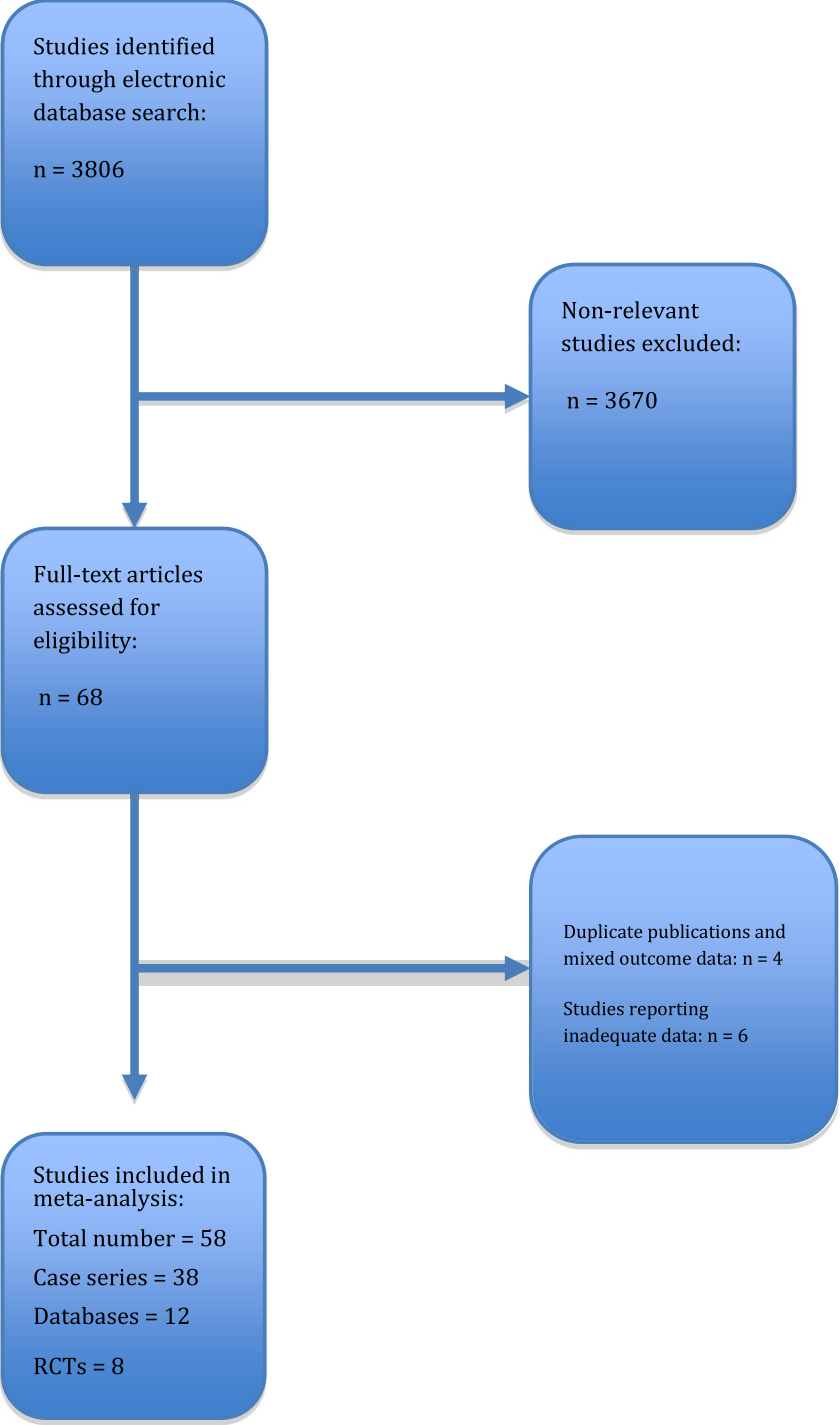
Literature search strategy. Flow chart showing the strategy used for the literature search

Our literature search yielded a total of 3806 unique articles on CEA of which 58 were eligible for this review (Fig. 1 shows the flow diagram of study selection for the analysis), totalling 8 RCTs, 12 databases and 38 case-series [1–3, 10, 11, 28–85]. 4 records had been excluded because they were series from the same institutions with duplicate clinical material. A further 6 articles had been excluded because they were reviews, or population-based studies from which accurate data could not be extracted. Another 3738 records were excluded because the title or the abstract were not relevant. This left 58 studies for analysis reporting data on sex and perioperative risk of CEA. The total number of CEAs included in our analysis was 468 045, of which 188 168 (40.2 %) were undertaken in women and the remaining 279 877 (59.8 %) CEAs were performed in male patients. Studies reporting CEAs were published between 1988 and 2014, whereas the patient recruitment period expanded from 1971 through 2013. The methodologic quality of the RCTs, represented in the Jadad score, was low. Similarly, a small proportion of the observational studies achieved a NOS score > 6 (15 of 58 studies). Main demographic and clinical features of the study populations are outlined in Table 1.

**Table 1:** Baseline characteristics of included studies

First author	Study type	Women (Total N)	Men (Total N)	Log odds ratio (stroke and mortality rate)	Std Err	Publication year	Asymptomatic women, %	Asymptomatic men, %	Dyslipidemia women, %	Dyslipidemia men, %	Hypertension women, %	Hypertension men, %	Diabetes women, %	Diabetes men, %	PAD women, %	PAD men, %	CAD women, %	CAD men, %	Smoker women, %	Smoker men, %	Age women, %	Age men, %	Shunt women, %	Shunt men, %	Redo CEA women, %	Redo CEA men, %	NOS, Jadad-score
Schneider 1997 [28]	CS/P	155	271	0,9887	0,6535	1997	30	23	-	-	74	68	16	22	-	-	39	53	59	58	72	71	28	28	-	-	
Rigdon 1998 [29]	CS/P	175	254	0,6951	0,4523	1998	-	-	-	-	-	-	-	-	-	-	-	-	-	-	-	-	-	-	-	-	
Akbari 2000 [30]	CS/P	520	778	−0,2442	0,3948	2000	51	44	-	-	77	66	42	36	-	-	40	50	53	68	71	70	-	-	-	-	
Ballotta 2000 [31]	CS/P	196	423	−0,4899	0,8064	2000	35	35	43	46	64	55	48	30	61	69	17	24	71	62	71	70	13	15	-	-	
Sternbach 2000 [33]	CS/P	68	88	0,2612	1,4235	2000	-	-	47	47	70	69	24	19	-	-	13	29	47	60	71	70	-	-	-	-	
Schneider 2000 [32]	CS/P	90	492	−0,7106	1,0508	2000	-	-	-	-	-	-	-	-	-	-	--	-	-	-	-	-	-	-	-	-	
James 2001 [36]	CS/P	125	199	0,4772	0,7165	2001	51	45	60	56	75	66	21	21	-	-	10	15	68	74	70	70	71	71	-	-	
Mattos 2001 [35]	CS/P	465	739	−0,9296	0,7927	2001	33	33	-	-	66	61	29	23	-	-	27	39	41	51	69	68	61	61	-	-	
Sarac 2002 [37]	CS/P	1148	2274	0,4837	0,2212	2002	74	74	-	-	-	-	23	22	-	-	57	73	-	-	69	69	-	-	7	6	
Lane 2003 [39]	CS/P	115	246	0,9757	0,6164	2003	50	58	17	29	75	70	17	25	-	-	10	8	58	68	73	71	33	30	-	-	
Lee 2003 [83]	CS/P	600	903	−0,1240	0,4020	2003	48	45	45	37	77	67	41	36	17	18	40	50	69	84	71	70	79	74	-	-	
Weise 2004 [84]	CS/P	56	156	0,5953	0,5927	2004	27	26	37	34	70	57	29	25	18	19	23	22	-	-	65	64	-	-	-	-	
Harthun 2005 [78]	CS/P	5950	8144	0,3474	0,1381	2005	-	-	-	-	77	77	8	6	-	-	28	40	-	-	70	70	-	-	-	-	
Hugl 2006 [77]	CS/P	115	229	−2,6675	1,4444	2006	84	76	-	-	-	-	-	-	-	-	-	-	-	-	-	-	-	-	-	-	
Park 2008 [75]	CS/P	40	53	−1,3691	1,5633	2008	52	53	-	-	93	94	30	23	-	-	23	17	70	70	70	72	-	-	-	-	
Dorigo 2009 [71]	CS/P	1200	2809	0,8562	0,4097	2009	66	64	32	27	73	64	22	19	25	34	15	23	36	81	72	71	-	-	3	4	
Poisson 2010 [65]	CS/P	52	84	−1,1718	1,1100	2010	69	61	44	53	72	71	25	42	10	10	27	40	77	91	76	73	-	-	-	-	
Yavas 2010 [66]	CS/P	42	163	−0,7495	1,0751	2010	38	35	41	22	52	41	21	22	14	31	15	19	12	34	64	65	69	70	-	-	
Baracchini 2012 [61]	CS/P	466	992	0,2461	0,7324	2012	32	36	60	52	66	59	29	34	53	60	40	45	63	69	76	75	18	14	-	-	
Luebke 2014 [58]	CS/P	588	1292	0,095	0,4113	2013	78	75	33	39	85	81	22	26	24	30	34	45	32	38	76	75	24	17	-	-	
Guzman 2013 [59]	CS/P	363	683	−0,1327	0,4624	2013	33	36	50	52	76	72	23	25	25	32	38	47	62	68	71	70	82	79	2	1	
Friedmann 1988 [40]	CS/S	280	408	−0,1776	0,3870	1988	-	-	-	-	-	-	-	-	-	-	-	-	-	-	-	-	-	-	-	-	
Maxwell 1990 [42]	CS/S	289	345	0,8866	0,6176	1990	-	-	-	-	-	-	-	-	-	-	-	-	-	-	-	-	-	-	-	-	
Magnan 1993 [43]	CS/S	90	300	0,1837	0,5354	1993	-	-	-	-	-	-	-	-	-	-	-	-	-	-	-	-	-	-	-	-	
Goldstein 1994 [44]	CS/S	256	441	−0,054	0,2840	1994	-	-	-	-	-	-	-	-	-	-	-	-	-	-	-	-	-	-	-	-	
Riles 1994 [41]	CS/S	844	1488	0,2052	0,2541	1994	-	-	-	-	-	-	-	-	-	-	-	-	-	-	-	-	-	-	-	-	
Plestis 1996 [45]	CS/S	396	610	0,8950	0,4322	1996	-	-	-	-	-	-	-	-	-	-	-	-	-	-	-	-	-	-	-	-	
Hertzer 1997 [46]	CS/S	652	1272	0,6340	0,3162	1997	60	60	-	-	-	-	-	-	-	-	-	-	-	-	-	-	-	-	-	-	
Kerdiles 1997 [47]	CS/S	103	178	−0,3499	0,4409	1997	-	-	-	-	-	-	-	-	-	-	-	-	-	-	-	-	-	-	-	-	
Goldstein II 1998 [80]	CS/S	151	312	1,2340	0,5790	1998	-	-	-	-	-	-	-	-	-	-	-	-	-	-	-	-	-	-	-	-	
Karp 1998 [49]	CS/S	910	1035	0,4606	0,2722	1998	-	-	-	-	-	-	-	-	-	-	-	-	-	-	-	-	-	-	-	-	
Kucey 1998 [48]	CS/S	434	847	0,3147	0,2342	1998	-	-	-	-	-	-	-	-	-	-	-	-	-	-	-	-	-	-	-	-	
Blohme 1999 [50]	CS/S	94	178	−0,4864	0,5920	1999	-	-	-	-	-	-	-	-	-	-	-	-	-	-	-	-	-	-	-	-	
Hartmann 1999 [51]	CS/S	46	62	0,5700	0,7011	1999	-	-	-	-	-	-	-	-	-	-	-	-	-	-	-	-	-	-	-	-	
Frawley 2000 [53]	CS/S	312	688	0,3915	0,5315	2000	5	5	-	-	-	-	-	-	-	-	-	-	-	-	68	68	-	-	-	-	
Naylor 2000 [54]	CS/S	171	329	0,4833	0,6130	2000	-	-	-	-	-	-	-	-	-	-	-	-	-	-	-	-	-	-	-	-	
Eckstein 2002 [55]	CS/S	45	119	−0,5647	0,8021	2002	-	-	-	-	-	-	-	-	-	-	-	-	-	-	-	-	-	--	-	-	
Dalainas 2007 [76]	CS/S	936	2396	0,5283	0,1894	2007	29	70	-	-	-	-	-	-	-	-	-	-	-	-	-	-	-	-	-	-	
McCrory 1993 [82]	DB	407	753	0,055	0,2411	1993	-	-	-	-	-	-	-	-	-	-	-	-	-	-	-	-	-	-	-	-	
Huber 1998 [81]	DB	19508	27233	0,095	0,067	1998	-	-	-	-	-	-	-	-	-	-	-	-	-	-	-	-	-	-	-	-	
Rockman 2001 [34]	DB	991	1485	−0,3863	0,5409	2001	34	30	-	-	62	54	20	23	-	-	38	50	36	38	69	69	31	30	-	-	
Tu 2003 [79]	DB	2096	3942	0,038	0,1127	2003	-	31	-	-	-	64	-	23	-	27	-	36	-	43	-	-	-	-	-	-	
Kapral 2003 [38]	DB	2096	3942	0,060	0,1131	2003	31	30	37	35	71	61	23	23	25	28	33	37	63	76	68	68	29	29	-	-	
Sidawy 2009 [72]	DB	551	817	−0,2644	0,1156	2009	40	60	-	-	-	-	-	-	-	-	-	-	-	--	-	-	-	-	-	-	
Halm 2009 (198–1999) [73]	DB	4125	5181	0,1419	0,1061	2009	-	-	-	-	-	-	-	-	-	-	-	-	-	-	-	-	-	-	-	-	
Rockman 2011 [64]	DB	21621	29162	−0,037	0,5856	2011	95	95	-	-	-	-	-	-	-	-	-	-	-	-	-	-	-	-	-	Blank	
Bisdas NYS 2012 [62]	DB	1969	3133	0,0676	0,1908	2012	92	91	45	44	74	71	29	29	-	-	34	45	-	-	72	71	-	-	-	-	
Menyhei 2012 [63]	DB	15358	32637	0,7540	0,5774	2012	32	68	-	-	-	-	-	-	-	-	-	-	-	-	-	-	-	-	-	-	
Jim 2013 [11]	DB	2678	3814	0,0241	0,1390	2013	61	58	-	-	85	83	31	32	45	42	40	54	57	63	71	71	-	-	10	8	
Kuy 2014 (NIS DATABASE) [10]	DB	94404	126849	0,008	0,036	2014	91	90	-	-	-	-	-	-	-	-	-	-	-	-	-	-	-	-	-	-	
ACAS 1995 [1]	RCT	281	544	0,7855	0,4654	1995	-	-	-	-	-	-	-	-	-	-	-	-	-	-	-	-	-	-	-	-	
ECST 1998 [3]	RCT	842	1962	0,5210	0,1932	1998	-	-	-	-	-	-	-	-	-	-	-	-	-	-	-	-	-	-	-	-	
NASCET 1998 [4]	RCT	424	1012	0,1660	0,2513	1998	-	-	-	-	-	-	-	-	-	-	-	-	-	-	-	-	-	-	-	-	
ACE 1999 [57]	RCT	842	1962	0,2067	0,1848	1999	-	-	-	-	-	-	-	-	-	-	-	-	-	-	-	-	-	-	-	-	
ACST 2004 [2]	RCT	469	936	0,4007	0,3249	2004	-	-	-	-	-	-	-	-	-	-	-	-	-	-	-	-	-	-	-	-	
CAVATAS 2009 [74]	RCT	75	178	−0,3873	0,3633	2009	-	-	-	-	-	-	-	-	-	-	-	-	-	-	-	-	-	-	-	-	
EVA-3 s, SPACE, ICSS [56, 69, 87, 98]	RCT	476	1232	0,2746	0,2203	2010	-	-	-	-	-	-	-	-	-	-	-	-	-	-	-	-	-	-	-	-	
CREST 2013 [87]	RCT	417	823	−0,1214	0,4059	2013	46	48	86	86	86	86	31	30	-	-	-	-	27	26	69	69	-	-	-	-	

### Overall analyses

#### Combined 30-day stroke and mortality rate

**Fig. 2:**
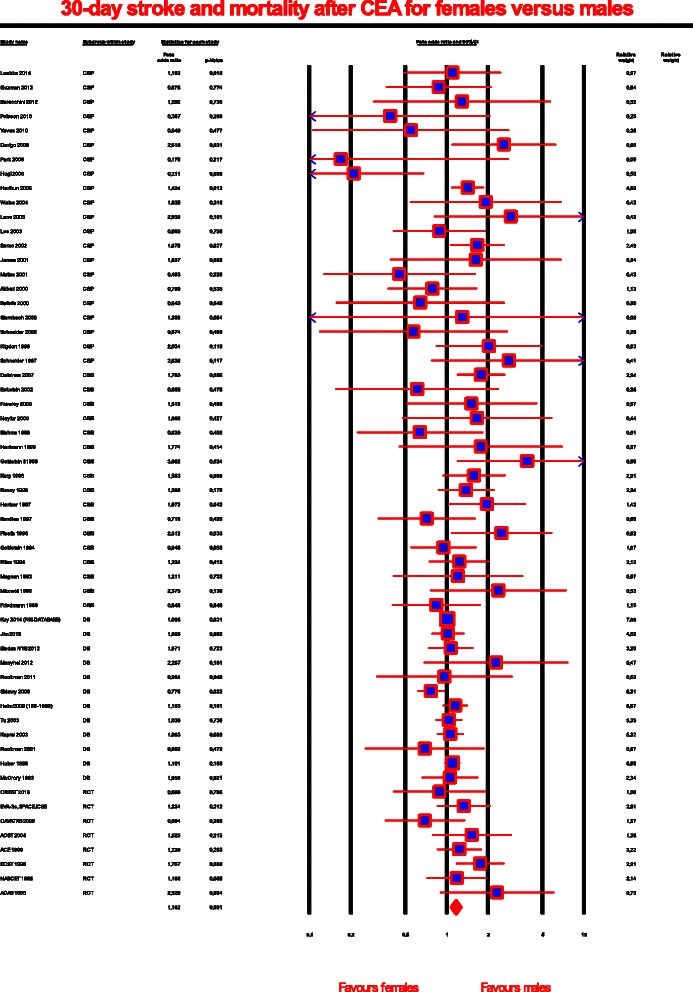
Forrest plot for carotid endarterectomy and stroke and death rates subjected to the gender. The odds for stroke and death within 30 days of CEA for females versus males. P is the statistical significance of the pooled Peto odds ratio. The size of the data marker indicates the weight of each trial. OR, odds ratio

All 58 articles reported combined stroke and mortality rates perioperatively or within 30 days of treatment (Fig. 2). The incidence of stroke and death in the male and female groups was 4 609/279 877 (1.6 %) and 3 254/188 168 (1.7 %), respectively, and this difference was statistically significant (Peto OR, 1,162; 95 % CI, 1.067-1.266; *P* = .001). Moderate heterogeneity among the studies was identified (*I*^2^ = 36 %), and the possibility of publication bias was low (*P* = .03).

#### 30-day stroke rate

**Fig. 3:**
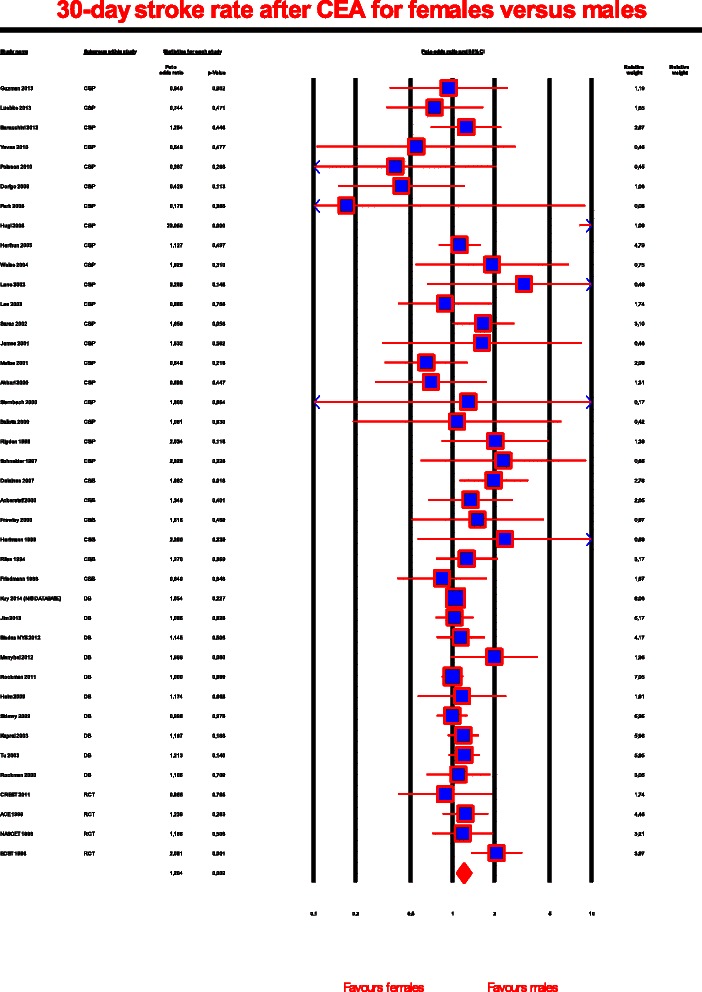
Forrest plot for carotid endarterectomy and stroke rates subjected to the gender. The odds for stroke within 30 days of CEA for females versus males. P is the statistical significance of the pooled Peto odds ratio. The size of the data marker indicates the weight of each trial. OR, odds ratio

40 studies reported 30-day stroke rates in both gender groups [3, 4, 10, 11, 29–32, 34–42, 52–54, 58–60, 62–67, 72–74, 76–80, 84–86] (Fig. 3). The overall stroke rate within 30 days of treatment for men and women were 2 916/242 494 (1.2 %) and 2 110/163 346 (1.3 %), respectively, and the difference between the groups was significant (Peto OR, 1.204; 95 % CI, 1.073-1.351; *P* = .002). Moderate heterogeneity among the studies existed (*I*^2^ = 47.9 %), and the likelihood of publication bias was low (*P* = .04).

### Sensitivity analyses

#### Combined 30-day stroke and mortality rate

##### Case series - primary aim

Among the 21 studies [29–34, 36–38, 40, 59, 62, 66, 67, 69, 72, 76, 78, 79, 84, 85] reporting combined 30-day stroke and mortality rates as their primary aim there was no difference in the association between sex and the combined end-point when applying the random-effects model (Peto OR, 1.202; 95 % CI, 0.925-1.561; *P* = .168).

However, when applying the fixed-effects model for the same subset of data, there was a statistically significant difference between the two genders regarding the combined end-point 30-day mortality and stroke rate (Peto OR, 1.299; 95 % CI, 1.089-1.548; *P* = .004). Moderate heterogeneity among the studies existed (*I*^2^ = 32.9 %), and the likelihood of publication bias was low (*P* = .23).

### Case series - secondary aim

Among the 17 [41–52, 54–56, 77, 81] studies reporting combined 30-day stroke and mortality rates as a subanalysis along with other risk factors or between case series there was a significant difference in the association between sex and the combined end-point, even when applying the random-effects model (Peto OR, 1.390; 95 % CI, 1.148-1.684; *P* = .001).

When applying the fixed-effects model for the same subset of data, there was a statistically significant difference between the two genders regarding the combined end-point 30-day mortality and stroke rate (Peto OR, 1.400; 95 % CI, 1.180-1.662; *P* < .000), as well. Moderate heterogeneity among the studies existed (*I*^2^ = 13.4 %), and the likelihood of publication bias was low (*P* = .82).

### Population based databases

Among the 12 database-studies [10, 11, 35, 39, 63–65, 73, 74, 80, 82, 83] reporting combined 30-day stroke and mortality rates as a secondary end-point there was no difference in the association between sex and the combined end-point when applying the random-effects model (Peto OR, 1.025; 95 % CI, 0.958-1.097; *P* = .474) as well as when using the fixed-effects model (Peto OR, 1.022; 95 % CI, 0.969-1.079; *P* = .419). Low heterogeneity among the studies existed (*I*^2^ = 11.2 %), and the likelihood of publication bias was low (*P* = .83).

### RCTs

Among the 10 RCTs [1–4, 56, 58, 69, 75, 86, 87] reporting combined 30-day stroke and mortality rates as a subanalysis along with other risk factors there was a significant difference in the association between sex and the combined end-point, even when applying the random-effects model (Peto OR, 1.302; 95 % CI, 1.060-1.600; *P* = .012).

When applying the fixed-effects model for the same subset of data, there was a statistically significant difference between the two genders regarding the combined end-point 30-day mortality and stroke rate (Peto OR, 1.310; 95 % CI, 1.089-1.576; *P* = .004), as well. Low heterogeneity among the studies existed (*I*^2^ = 15 %), and the likelihood of publication bias was low (*P* = .69).

### All study types with gender analyses as a secondary aim

When combining all 38 [1–4, 10, 11, 35, 39, 41–52, 54–56, 58, 63–65, 69, 73–75, 77, 80–83, 86, 87] studies with gender analyses as a secondary aim, random-effects meta-analysis as well as fixed-effects meta-analysis reveal a significant association between sex and the combined end-point (Peto OR, 1.150; 95 % CI, 1.050-1.260; *P* = .003 and Peto OR, 1.068; 95 % CI, 1.017-1.123; *P* = .009, respectively). Moderate heterogeneity among the studies existed (*I*^2^ = 36.1 %), and the likelihood of publication bias was low (*P* = .61).

### Studies published after the year 2004

Among the 24 studies [2, 10, 11, 56, 59, 60, 62–67, 69, 72–79, 85–87] published after the year 2004 and reporting combined 30-day stroke and mortality rates there was no difference in the association between sex and the combined end-point when applying the random-effects model (Peto OR, 1.119; 95 % CI, 0.983-1.274; *P* = .088) as well as when using the fixed-effects model (Peto OR, 1.043; 95 % CI, 0.984-1.105; *P* = .155). Moderate heterogeneity among the studies existed (*I*^2^ = 45.5 %), and the likelihood of publication bias was low (*P* = .38).

The combined outcome estimate of combined 30-day mortality and stroke rates was not substantially affected when the primary analysis was repeated with a fixed-effects model (OR, 1.084; 95 % CI, 1.033-1.137; P = .001), altered data sets after excluding each single study at a time (OR, 1.177; 95 % CI, 1.076-1.297; *P* < .01), or cumulative analysis (OR, 1.177; 95 % CI, 1.076-1.2879; *P* < .01).

### 30-day stroke rate

#### Case series - primary aim

Among the 20 [29–32, 34, 36–38, 40, 59, 60, 62, 66, 67, 72, 76, 78, 79, 84, 85] studies reporting 30-day stroke rates as their primary aim there was no difference in the association between sex and the end-point when applying the random-effects model (Peto OR, 1.322; 95 % CI, 0.922-1.895; *P* = .129).

However, when applying the fixed-effects model for the same subset of data, there was a statistically significant difference between the two genders regarding the end-point 30-day stroke rate (Peto OR, 1.235; 95 % CI, 1.024-1.490; *P* = .027). Moderate heterogeneity among the studies existed (*I*^2^ = 63.4 %), and the likelihood of publication bias was low (*P* = .44).

#### Case series - secondary aim

Among the 6 studies [41, 42, 52–54, 77] reporting 30-day stroke rates as a subanalysis along with other risk factors or between case series there was a significant difference in the association between sex and the end-point, even when applying the random-effects model (Peto OR, 1.403; 95 % CI, 1.052-1.871; *P* = .021).

When applying the fixed-effects model for the same subset of data, there was a statistically significant difference between the two genders regarding the end-point 30-day stroke rate (Peto OR, 1.403; 95 % CI, 1.052-1.871; *P* = .021), as well. No heterogeneity among the studies existed (*I*^2^ = 0 %), and the likelihood of publication bias was low (*P* = .71).

#### Population based databases

Among the 10 database-studies [10, 11, 35, 39, 63–65, 73, 74, 80] reporting 30-day stroke rates as a secondary end-point there was no difference in the association between sex and the end-point when applying the random-effects model (Peto OR, 1.060; 95 % CI, 0.992-1.133; *P* = .086) as well as when using the fixed-effects model (Peto OR, 1.060; 95 % CI, 0.992-1.133; *P* = .086). No heterogeneity among the studies existed (*I*^2^ = 0 %), and the likelihood of publication bias was low (*P* = .12).

#### RCTs

Among the 4 RCTs [3, 4, 58, 86] reporting 30-day stroke rates as a subanalysis along with other risk factors there was no difference in the association between sex and the end-point, even when applying the random-effects model (Peto OR, 1.364; 95 % CI, 0.979-1.901; *P* = .067).

However, when applying the fixed-effects model for the same subset of data, there was a statistically significant difference between the two genders regarding the end-point 30-day stroke rate (Peto OR, 1.398; 95 % CI, 1.106-1.765; *P* = .005), as well. Moderate heterogeneity among the studies existed (*I*^2^ = 45.8 %), and the likelihood of publication bias was low (*P* = .73).

#### All study types with gender analyses as a secondary aim

When combining all 20 studies [3, 4, 10, 11, 35, 39, 41, 42, 52–54, 58, 63–65, 73, 74, 77, 80, 86] with gender analyses as a secondary aim, random-effects meta-analysis as well as fixed-effects meta-analysis reveal a significant association between sex and the end-point (Peto OR, 1.140; 95 % CI, 1.040-1.249; *P* = .005 and Peto OR, 1.096; 95 % CI, 1.029-1.167; *P* = .004, respectively). Low heterogeneity among the studies existed (*I*^2^ = 18.9 %), but the likelihood of publication bias was high (*P* = .02).

#### Studies published after the year 2004

Among the 19 studies [10, 11, 59, 60, 62–67, 72–74, 76–79, 85, 86] published after the year 2004 and reporting 30-day stroke rates there was no difference in the association between sex and the end-point when applying the random-effects model (Peto OR, 1.182; 95 % CI, 0.989-1.414; *P* = .066). However, when using the fixed-effects model (Peto OR, 1.071; 95 % CI, 1.002-1.144; *P* = .043) a significant difference was revealed. Moderate heterogeneity among the studies existed (*I*^2^ = 65.4 %), and the likelihood of publication bias was low (*P* = .21).

The combined outcome estimate of combined 30-day mortality and stroke rates was not substantially affected when the primary analysis was repeated with a fixed-effects model (OR, 1.109; 95 % CI, 1.045-1.177; *P* = .001), altered data sets after excluding each single study at a time (OR, 1.216; 95 % CI, 1.077-1.373; *P* = .002), or cumulative analysis (OR, 1.216; 95 % CI, 1.077-1.373; *P* = .002).

### Meta-regression analyses

Meta-regression analysis investigated potential effects of clinical confounders on perioperative adverse events associated with CEA subjected to the gender.

#### Year of publication

**Fig. 4:**
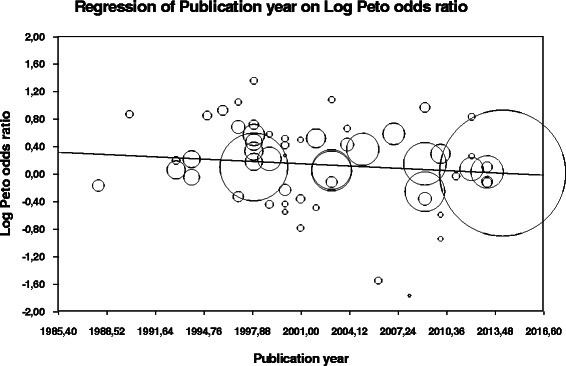
Scatterplot of the relationship between year of publication and Log Odds Ratio for stroke and death. Scatterplot shows the relationship between year of publication and log odds ratio for stroke and mortality

Random-effects meta- regression revealed a statistical significant evidence for an association between the log OR for 30-day stroke and mortality and the year of publication (*p* = .004) (Fig. 4). This underlines that stroke and mortality rates have reduced over time and that there is a significant association (slope coefficient (s.e.) = −0.01105 (0,00344), *p* = .00127).

#### Mean age

Details on mean age were available in 24 studies (availability of information: 60 832/468 045 patients, 12.9 %). Metaregression showed no statistically significant association between mean age and 30-day stroke and mortality (slope coefficient (s.e.) = 0.00972 (0.03199), *p* = .532).

#### Arteria hypertension

Details on arterial hypertension were available in 23 studies (availability of information: 54 314/468 045 patients, 11.6 %). Metaregression showed no statistically significant association between arterial hypertension and 30-day stroke and mortality (slope coefficient (s.e.) = 0.00310 (0.00661), *p* = .582).

#### Diabetes mellitus

Details on diabetes mellitus were available in 24 studies (availability of information: 57 736/468 045 patients, 12.3 %). Metaregression showed no statistically significant association between diabetes mellitus and 30-day stroke and mortality (slope coefficient (s.e.) = −0.01472 (0.00655), *p* = 0.482).

#### Coronary artery disease

Details on coronary artery disease were available in 24 studies (availability of information:56 496/468 045 patients, 12.1 %). Metaregression documented no statistically significant association between coronary artery disease and 30-day stroke and mortality (slope coefficient (s.e.) = −0.00134 (0.00512), *p* = 0.445).

#### Peripheral arterial disease

Details on peripheral artery disease were available in 12 studies (availability of information:27 540/468 045 patients, 5.9 %). Metaregression documented no statistically significant association between coronary artery disease and 30-day stroke and mortality (slope coefficient (s.e.) = −0.00313 (0.00932), *p* = 0.726).

#### Dyslipidemia

Details on dyslipidemia were available in 15 studies (availability of information: 24 289/468 045 patients, 5.2 %). Metaregression showed no statistically significant association between dyslipidemia and 30-day stroke and mortality (slope coefficient (s.e.) = −0.00691 (0.00735), *p* = 0.801).

#### Smoking status

Details on smoking status were available in 20 studies (availability of information: 34 906/468 045 patients, 7.4 %). Metaregression showed no statistically significant association between smoking status and 30-day stroke and mortality (slope coefficient (s.e.) = 0.00593 (0.00579), *p* = 0.677).

#### Symptom status

Details on symptom status were available in 30 studies (availability of information: 371 485/468 045 patients, 79.4 %). Metaregression showed a statistically significant association between symptom status and 30-day stroke and mortality (slope coefficient (s.e.) = −0.00049 (0.00153), *p* = 0.00893).

### Quality assessment

The methodologic quality of the 10 RCTs included in the present meta-analysis, represented in the Jadad score, was low (all studies: Jadad score 3). Similarly, only a small proportion of the observational studies achieved a NOS score > 6 (15 of 58 studies).

### TSA for 30-day stroke and mortality rate

For the outcome of 30-day death or stroke, the required diversity was calculated based on an RRI of 20 %, alpha of 5 %, and beta of 20 %.

#### Studies published after the year 2004

**Fig. 5:**
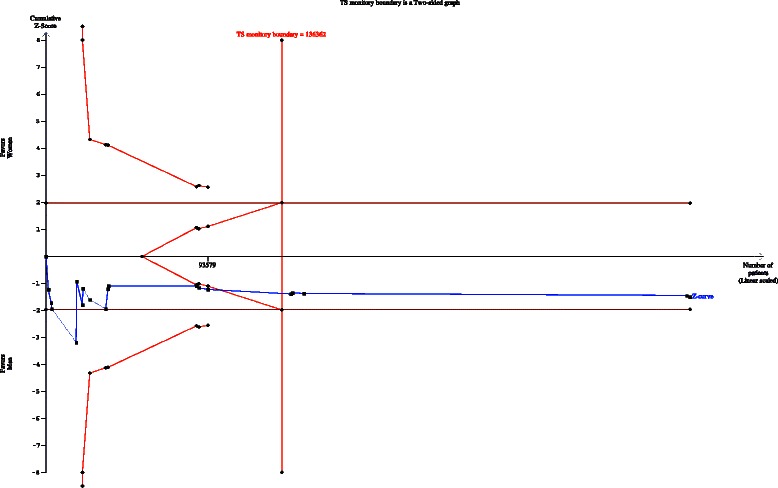
Trial sequential analysis of the effects of carotid endarterectomy on periprocedural stroke and death subjected to the gender and publication date after the year 2004

The cumulative z curve crossed both the traditional boundary and the TSMB for the outcomes of death or stroke, demonstrating firm evidence for a 20 % RRI in the female group compared with the male group (Fig. 5).

#### Case series – primary aim

**Fig. 6:**
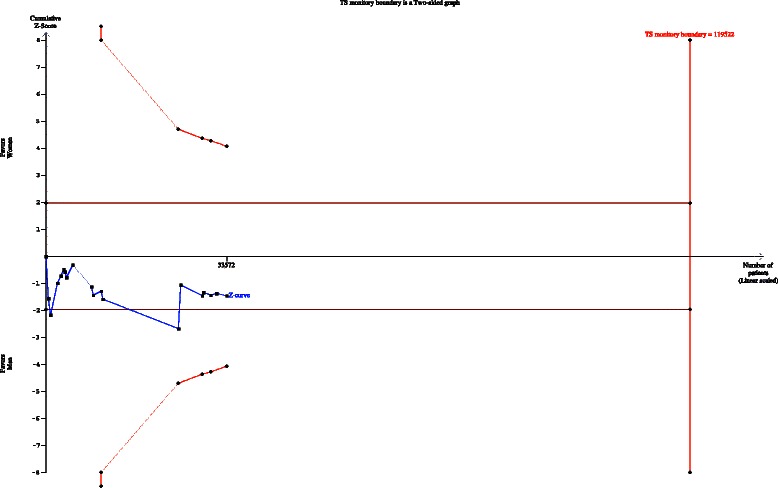
Trial sequential analysis of the effects of carotid endarterectomy on periprocedural stroke and death subjected to the gender and type of study (outcome of interest as primary aim)

The cumulative z curve crossed the traditional boundary but not the TSMB, suggesting a lack of firm evidence for an RRI of 20 % in the female group compared with the male group (Fig. 6).

#### Case series – secondary aim

**Fig. 7:**
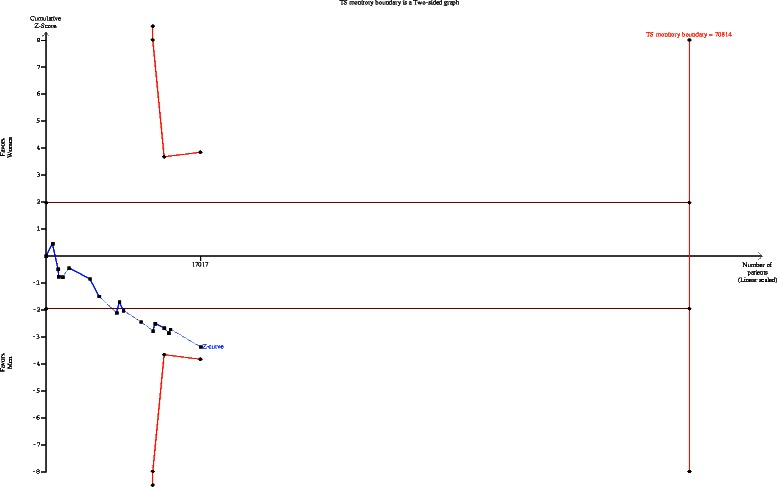
Trial sequential analysis of the effects of carotid endarterectomy on periprocedural stroke and death subjected to the gender and type of study (outcome of interest as secondary aim)

The cumulative z curve crossed the traditional boundary but not the TSMB, suggesting a lack of firm evidence for an RRI of 20 % in the female group compared with the male group (Fig. 7).

#### RCTs

The cumulative z curve crossed the traditional boundary but not the TSMB, suggesting a lack of firm evidence for an RRI of 20 % in the female group compared with the male group.

## Discussion

Gender-based outcomes and the etiology of sex-related mechanisms in patients treated with CEA are a topic of considerable debate in the recent vascular literature [34, 35, 38, 39, 64, 71, 77, 88]. Till now, there are no generally accepted and obliging guidelines regarding the preoperative selection of CEA patients subjected to the gender [59, 89, 90].

### RCT data

Subgroup analysis of the large CEA-randomised trials have suggested that the benefit from CEA would be less in women compared to men for both, symptomatic and asymptomatic carotid stenosis. In particular, in the European Carotid Surgery Trial (ECST) and the Asymptomatic Carotid Surgery Trial (ACST) women appeared to have higher risks of perioperative complications, and in the moderate (50 % to 69 %) stenosis arm of the North American Symptomatic Carotid Endarterectomy Trial (NASCET) women did not appear to benefit from surgery at all. In this subset of female patients CEA was associated with a marginal or reduced long-term benefit combined with at least 2-fold increase in the perioperative complication rate as compared to men. It has been hypothesized, that the lower degree of benefit in women was caused partly by a (slightly) higher operative risk in combination with a lower natural history risk of stroke in women as compared to men [5–7]. However, these assumptions are based on post-hoc subgroup analyses on results of these RCTs and have therefore some inherent limitations. Women comprised only a third of patients in these trials (between 28 and 34 %) and this relatively small sample size has left them underpowered to detect important differences between women and men. In addition to that, selection criteria for these randomized controlled trials may partly explain the discrepant findings and thus limiting their generalizability to the typical endarterectomy population. As a result, these gender-related results are difficult to interpret due to significant design limitations [5] and due to a lack of adjustment for other important prognostic factors. In the recently published International Carotid Stenting Study (ICSS), women had a higher 120-day event rate for CEA compared to men (7.6 % vs 4.2 %) [91]. On the contrary, in the Carotid Revascularization Endarterectomy vs Stenting Trial (CREST), women undergoing CEA had a lower periprocedural event rate compared to men (3.8 % vs 4.9 %) [87].

The present unselected meta-analysis of RCTs revealed that female patients undergoing CEA had a higher combined risk of death and stroke after the intervention than did male patients (Peto OR, 1,162; 95 % CI, 1.067-1.266; *P* = .001). We analyzed the robustness of the observed outcomes by performing sensitivity analyses. Low heterogeneity among the studies existed (*I*^2^ = 15 %), and the likelihood of publication bias was low (*P* = .69). In the TSA, the cumulative z curve crossed the traditional boundary but not the TSMB, suggesting a lack of firm evidence for an RRI of 20 % in the female group compared with the male group in RCTs for the endpoint under investigation. As a result, the higher perioperative risk of stroke and death after CEA in women observed in large RCTs is likely to be a statistical artifact due to small female patient numbers. In addition to that, because of low absolute difference between the gender related outcomes (Peto OR, 1,162; 95 % CI, 1.067-1.266; *P* = .001), the clinical significance of this finding is unclear. As well, we found a significant difference in overall perioperative stroke rates for CEA when men were compared with women (Peto OR, 1.204; 95 % CI, 1.073-1.351; *P* = .002). Sensitivity analysis revealed that there might be a statistically significant difference between the two genders regarding the end-point 30-day stroke rate by using the fixed-effects model, as well (Peto OR, 1.398; 95 % CI, 1.106-1.765; *P* = .005), but again the clinical significance of this small difference may be narrow. In this sensitivity analysis, even moderate heterogeneity among the studies existed (*I*^2^ = 45.8 %) but the likelihood of publication bias was low (*P* = .73).

It has also been suggested that the RCT results may be confounded by referral bias [86]. Women are less likely than men to be selected for both, cardiac and peripheral vascular surgery [92] and recent studies have demonstrated that women are less likely to receive CEA or angioplasty than men in the same situation [86]. Therefore, it might be possible that those women who actually undergo surgery are at higher risk of complications than those who do not. As a result, it is unclear whether these RCT results can be generalised to the non-trial setting [93, 94]. It is therefore essential to analyze whether the RCT results of CEA are also seen in routine clinical practice.

### Non-RCT data

#### Sex related differences as a primary aim of the study

The case series reporting combined 30-day stroke and mortality rates subjected to the sex as their primary aim found CEA as safe and beneficial in women as in men when applying the random-effects model (Peto OR, 1.202; 95 % CI, 0.925-1.561; *P* = .168). However, when applying the fixed-effects model for the same subset of data, there was a statistically significant difference between the two genders regarding the combined end-point 30-day mortality and stroke rate (Peto OR, 1.299; 95 % CI, 1.089-1.548; *P* = .004). Moderate heterogeneity among the studies existed (*I*^2^ = 32.9 %), and the likelihood of publication bias was low (*P* = .23). However, in TSA the cumulative z curve crossed the traditional boundary but not the TSMB, suggesting a lack of firm evidence for an RRI of 20 % in the female group compared with the male group in case series for the endpoint under investigation and gender differences as their primary aim. Thus, although those studies have shown no difference in perioperative stroke and mortality between men and women in the random effects model, criticism may be pointed to the size and power of those studies, raising the possibility of a type II error as demonstrated by TSA. The same was true when analyzing the endpoint 30-day stroke in case series with sex-related differences as a primary aim, with no difference in the association between sex and the end-point when applying the random-effects model (Peto OR, 1.322; 95 % CI, 0.922-1.895; *P* = .129), but a statistically significant difference between the two genders regarding the end-point 30-day stroke rate when applying the fixed-effects model (Peto OR, 1.235; 95 % CI, 1.024-1.490; *P* = .027). Moderate heterogeneity among the studies existed (*I*^2^ = 63.4 %), and the likelihood of publication bias was low (*P* = .44).

#### Sex-related differences as a secondary aim of the study

The case series reporting combined 30-day stroke and mortality rates subjected to the gender as a secondary aim suggest that CEA is associated with significantly increased risk for periprocedural death and stroke in women when compared with men when applying the random-effects model and the fixed-effects model, respectively (Peto OR, 1.390; 95 % CI, 1.148-1.684; *P* = .001, and Peto OR, 1.400; 95 % CI, 1.180-1.662; *P* < .000, respectively). Moderate heterogeneity among the studies existed (*I*^2^ = 13.4 %), and the likelihood of publication bias was low (*P* = .82). However, the cumulative z curve crossed the traditional boundary but not the TSMB, suggesting a lack of firm evidence for an RRI of 20 % in the female group compared with the male group in case series for the endpoint under investigation and gender differences as a secondary aim. Thus, although those studies have shown a difference in perioperative stroke and mortality between men and women in the random-effects and the fixed effects model, criticism may be pointed to the size and power of those studies, raising the possibility of a type II error as demonstrated by TSA. The same was true when analyzing the endpoint 30-day stroke in case series with gender differences as a secondary aim, with a significant difference in the association between sex and the end-point when applying the random-effects model (Peto OR, 1.403; 95 % CI, 1.052-1.871; *P* = .021) and the fixed-effects model (Peto OR, 1.403; 95 % CI, 1.052-1.871; *P* = .021), as well. No heterogeneity among the studies existed (*I*^2^ = 0 %), and the likelihood of publication bias was low (*P* = .71).

#### Databases

We found no difference in overall perioperative stroke rates and combined death and stroke rates for CEA when men were compared with women in database analyses. In extensive sensitivity analyses, we demonstrated the robustness of all observed outcomes under investigation. Low heterogeneity among the studies existed, and the likelihood of publication bias was low for all comparisons. Although registries and state-wide databases lack the granular details of patient demographics, comorbidities and procedure preferences, they represent large diverse populations without the institutional selection bias. Therefore, presumably, these databases reflect routine real-world medical practice as compared to databases from randomised controlled trials that usually include tertiary care and university centres only with carefully defined patient selection criteria and practitioner credentialing. These findings probably suggest that medical, daily, population wide practice is rather different from that in large centres.

#### Metaregression

Meta-regression analysis investigated potential effects of publication date of each study, age, hypertension, diabetes mellitus, coronary artery disease, peripheral artery disease, dyslipidemia, smoking status, and symptomatic or asymptomatic carotid disease on perioperative adverse events associated with CEA subjected to the gender. An interesting finding of the meta-regression analysis is that only the category “year of publication” and “symptom status” were significant confounders for the log odds ratio for stroke and mortality in male and female CEA patients. This essentially means that in older studies, the difference in stroke or mortality is large, becoming less as the years pass, and this reduction in difference is statistically significant. These results are in concordance with the study by Rockman et al. [64]. When stratified by the presence of preoperative symptoms, asymptomatic male and female patients undergoing carotid intervention had a nearly identical rate of postoperative stroke and in-hospital death. However, symptomatic women undergoing carotid artery interventions had a significantly higher rate of postoperative stroke than symptomatic men (3.8 % vs 2.3 %, *P* = .03). The clinical significance of this finding is unclear. It is possible, of course, that symptomatic female patients would be at a higher risk for future stroke if no intervention were performed and still benefit from intervention vs medical management.

### Own results

A former meta-analysis of the existing literature performed in 2005 by Bond et al. [95] found that women undergoing CEA did have a higher rate of operative stroke and death than men (odds ratio, 1.31; 95 % confidence interval, 1.17-1.47, *P* < .001). In the present meta-analysis, the effect of sex on the operative risk of CEA in case series was consistent with those observed in the RCTs. Given the potential concerns about the generalisability of observations made in trials to routine clinical practice, it was important to determine whether the increased operative risk of stroke and death in women observed in the trials of CEA were likely to be seen in routine clinical practice. In contrast to the results presented by Bond et al. [95], we have shown that the effects of sex on the operative risk of CEA in published series from routine clinical practice are not consistent with those observed in the RCTs and even differ between cases series with gender considerations as primary aim and those with gender aspects as a secondary aim and database analyses. Whereas the unselected overall meta-analysis, and the meta-analysis of case series with gender aspects as a secondary outcome showed a significantly increased risk for 30-day stroke and death in women compared to men, meta-analysis of databases and case series with sex-related outcomes as a primary aim demonstrated no increase in operative risk of stroke and death in women compared to men. As a result, the findings of reports in which the gender association was the primary subject of study were highly consistent with those in large databases in which the gender observations was one of many associations reported. Our unselected analysis found significant differences in overall stroke and mortality outcomes between women and men after CEA. In addition, there were also a differences found in stroke and mortality among asymptomatic and symptomatic patients from both sexes. These results do not support the generalisability of the analyses of the overall effects of CEA from the unselected study data to routine clinical practice.

Over the decades 1980 and 2015 optimal medical treatment has been changed tremendously. There is moderate strength of evidence among three quality-A randomized controlled trials (RCTs) (the Veterans Affairs Cooperative Study [VA], the Asymptomatic Carotid Atherosclerosis Study (ACAS), and the Asymptomatic Carotid Atherosclerosis Trial [ACST]) that carotid endarterectomy (CEA) and best medical therapy (BMT) can reduce the risk of ipsilateral stroke as compared with best medical therapy alone, which was demonstrated by all three trials. However, the results from these trials are not applicable to contemporary clinical practice, as they do not compare CEA with contemporary best medical therapy and under conditions of real-world adherence and persistence, respectively. Surgeons in contemporary clinical trials with up-to-date best medical treatment have also achieved CEA periprocedural death and stroke rates lower than those in pivotal trials. For example, in the Carotid Revascularization Endarterectomy vs. Stenting Trial (CREST), the death/stroke rates for symptomatic patients was 3.2 % and for asymptomatic patients was 1.4 %. To date, there is no RCT that has analysed the impact of contemporary best medical treaetment of carotid artery stenosis subjected to the sex as a primary aim of the study.

In the present study, among the 24 studies [2, 10, 11, 56, 59, 60, 62–67, 69, 72–79, 85–87] published after the year 2004 with a contemporary best medical treatment and reporting combined 30-day stroke and mortality rates there was no difference in the association between sex and the combined end-point when applying the random-effects model as well as when using the fixed-effects model. In addition to that, TSA showed that confidence can be put into these results. Although, the studies published after the year 2004 represent a more contemporary management of patients with carotid artery stenosis, the interpretation of these results regarding the impact of best medical treatment alone on these sex-stratified outcomes should be done with caution.

#### Possible reasons for gender differences

The reasons for the postulated perioperative risk difference remain speculative and the overall evidence for outcome differences by sex-specific characteristics is limited in the literature. Potential explanations for higher surgical risks in women include the older age of onset of cerebrovascular disease in women [96]. Other explanations for the sex disparities in benefit from CEA may be attributed to further reasons listed in Table 2 [97–105].

**Table 2:** Possible reasons for gender differences for carotid endarterectomy

	Possible reasons for gender differences	Reference
Epidemiology		
	Older age of onset of cerebrovascular disease in women	[97]
Plaque characteristics		
	Higher rates of carotid artery stenosis,	[97]
	Lower and more stable plaque burden for the same degree of carotid artery stenosis in females compared with males	
Anatomy		
	Female carotid arteries are higher- velocity vessels with increased outflow/inflow ratio,	[97–100]
	Women tend to have atherosclerotic plaque relatively localized and mainly distributed in the common carotid as opposed to the proximal internal carotid artery, usually seen in men	
	Smaller ICA size in women that might in turn lead to a higher incidence of early (immediate thrombosis, postoperative microembolization) and late (recurrent stenosis, ipsilateral stroke) postoperative complications	[78, 101–105]
Surgery		
	Higher surgical risk in women	[97]
Pathophysiology		
	Lower cerebrovascular reserves in women, as cerebrovascular reactivity after hypercapnia was found to be more impaired in postmenopausal women compared with men of the same age	[97–100]

### Limitations

Although we believe that our results are likely to be valid, our study does have some potential shortcomings. First, the studies included in the review were of varying methodological quality. Some were retrospective and only a minority of the remainder had independent assessment of outcome by a neurologist. However, although the absolute operative risk will therefore have been underestimated in some studies, this should not have biased the within-study relative odds of stroke and death due to surgery by sex. However, the reliability of the meta-analyses of the within-study comparisons is supported by the consistent results, with very little statistical heterogeneity between studies, in the present review. The same argument applies to the fact that the use of ancillary treatments, such as the use of patching, shunting or local anaesthetic, will also have varied between studies. Secondly, publication bias is a potential problem with analyses of published data. It is possible that some of the studies looked at the interaction of several risk factors with operative risk, but only published those that were ‘interesting’ or statistically significant. However, funnel plots did not show any obvious skewing suggestive of publication bias (data not shown) and the lack of heterogeneity between studies indicates that selective reporting of extreme results (either associations with low operative risk or high operative risk) was uncommon. Adequate power is difficult to achieve in institutional or even multicenter studies to make meaningful comparisons of rare events, but our analysis has an advantage in that a large patient cohort was used to calculate pooled outcome estimates, such as mortality and stroke. As in other meta-analyses, given the lack of data in each trial, we did not adjust our analyses for medications used during and following the procedure. Although detailed sensitivity analyses on many variables were undertaken, given heterogeneity in the study protocols, clinically relevant differences could have been missed and are best assessed in a meta-analysis of individual patient data. The subgroup analyses might suffer from multiple testing. As a result, the results of the sensitivity analyses are best described as secondary and hypothesis generating only. In addition to that, our report is limited by the heterogeneous groups of patients entering the meta- analysis models. No adjustments for differences in clinical characteristics of the study populations, such as presenting symptom status and atherosclerotic comorbidity, could be made. Further more, each of these studies reported the results of operations performed by multiple surgeons and, in the latter case, in multiple institutions. As a result, there was no standardization of the surgical approach with respect to the method of anesthesia, the use and method of cerebral monitoring, the use of an indwelling shunt, closure of the arteriotomy with a patch, and other factors. These variables were also not controlled in the ACAS [5] and could potentially confound an analysis of the impact of gender on surgical outcome.

## Conclusions

In conclusion, we have shown that the effects of sex on the operative risk of CEA in published series from routine clinical practice are not consistent with those observed in the RCTs and even differ between cases series with gender considerations as primary aim and those with gender aspects as a secondary aim and database analyses. Whereas the unselected overall meta-analysis, and the meta-analysis of case series with gender aspects as a secondary outcome showed a significantly increased risk for 30-day stroke and death in women compared to men, meta-analysis of databases and case series with gender related outcomes as a primary aim demonstrated no increase in operative risk of stroke and death in women compared to men. This highlights the need for more sex-specific trials that will provide solid information regarding the management of carotid disease in women, define procedural indications for different risk groups, and provide clear guidelines for the community.
